# Leflunomide abrogates neuroinflammatory changes in a rat model of Alzheimer’s disease: the role of TNF-α/NF-κB/IL-1β axis inhibition

**DOI:** 10.1007/s00210-022-02322-3

**Published:** 2022-11-17

**Authors:** Menna Nafea, Mona Elharoun, Mohammad Mohmoud Abd-Alhaseeb, Maged Wasfy Helmy

**Affiliations:** 1 Department of Pharmacology and Biochemistry, College of Pharmacy, Arab Academy for Science, Technology & Maritime Transport, Alexandria, Egypt; 2 Department of Pharmacology and Toxicology, Faculty of Pharmacy, Damanhour University, Damanhour, El-Bahira Egypt

**Keywords:** Alzheimer’s disease, Leflunomide, Aluminum chloride, Hippocampus, Neuroinflammation, Cholinergic activity

## Abstract

Alzheimer’s disease (AD) is one of the most common neurodegenerative diseases and is associated with disrupted cognition and behavior. Neuroinflammatory pathogenesis is the main component that contributes to AD initiation and progression through microglial activation and neuronal damage. Thus, targeting inflammatory pathways may help manage AD. In this study, for the first time, the potential prophylactic and therapeutic effects of leflunomide were investigated either alone or in combination with rivastigmine in aluminum chloride (AlCl_3_)-induced AD-like rats using behavioral, biochemical, and histological approaches. Thirty-six adult male albino rats were divided into two protocols: the treatment protocol, subdivided into five groups (*n* = 6)—(1) control group, (2) AlCl_3_ (50, 70, 100 mg/kg/I.P) group, (3) reference group (rivastigmine 2 mg/kg/P.O.), (4) experimental group (leflunomide 10 mg/kg/P.O.), and (5) combination group (rivastigmine + leflunomide); and the prophylactic protocol (leflunomide 10 mg/kg/P.O.), which started 2 weeks before AlCl_3_ induction. The results showed that AlCl_3_ disrupted learning and memory parameters in rats and increased amyloid-β plaque deposition and neurofibrillary tangle aggregation. Moreover, AlCl_3_ administration markedly elevated acetylcholinesterase activity, nuclear factor-kappa β, tumor necrosis factor-α, and interleukin-1 beta, and marked degenerative changes in the pyramidal neurons. However, administration of leflunomide alone or with rivastigmine in AlCl_3_-induced AD rats restored most of the behavioral, biochemical, and histological parameters triggered by AlCl_3_ in rats. Our findings suggest that leflunomide can potentially restore most of the neuronal damage in the hippocampal tissues of AlCl_3_-induced AD rats. However, these preclinical findings still need to be confirmed in clinical trials.

**Supplementary information:**

The online version contains supplementary material available at 10.1007/s00210-022-02322-3.

## Introduction

AD is rapidly becoming one of the world’s most serious cognitive diseases (Sinyor et al. 2020). Globally, over 50 million people are diagnosed with AD, and this number is expected to exceed 152 million by the year 2050 (Shunan et al. 2021). Accumulation and deposition of amyloid beta (Aβ) peptides and neurofibrillary tangles are the significant hallmarks of AD. However, neuroinflammation has recently emerged as a third feature of the disease (Heneka et al. 2014). Pro-inflammatory cytokines play an important role in the AD development (Anuradha et al. 2022). Chronic deposition of Aβ in the brain promotes cerebral neuroinflammation by activating the microglia, which are thought to be a major source of pro-inflammatory cytokines in AD (Prinz et al. 2011). Aβ binding to the surface of microglial cells induces pro-inflammatory gene expression and increases in pro-inflammatory cytokines such as tumor necrosis factor-alpha (TNF-α), interleukin (IL)-1β, IL-6, and IL-18. These cytokines lead to tau hyperphosphorylation and neuronal loss (von Bernhardi et al. 2010).

Nuclear factor-kappa beta (NF-κβ) is one of the most important regulators of pro-inflammatory gene expression (Tak and Firestein 2001). It also regulates the synthesis of cytokines such as TNF-α, IL-1β, IL-6, and IL-8 (Aupperle et al. 1999). Central nervous system (CNS) dysfunction, oxidative stress, and neuroinflammation are the critical events activated in AD and potentiated by NF-κβ overexpression (Rather et al. 2021). Furthermore, several studies (Holmes et al. 2009; Perry et al. 2007) suggested that chronic inflammation from the periphery can induce pro-inflammatory cytokines in the CNS by crossing the blood–brain barrier (BBB) and contribute to cognitive decline in AD patients.

Currently, there is no efficient treatment or prevention of AD, and the current available remedies have moderate efficacy, only treating symptoms. The only four medications approved by the US Food and Drug Administration for AD are acetylcholinesterase (AChE) inhibitors (donepezil, galantamine, and rivastigmine) and NMDA antagonists (memantine) (Cummings et al. 2022). Thus, investigating additional medications for more effective AD treatment is urgently required (Du et al. 2018). Extensive efforts currently focus on treating inflammation in the AD development and progression. Targeting microglia pro-inflammatory cytokine production in AD by using anti-inflammatory and immunomodulatory drugs could offer a promising treatment modality for AD.

Leflunomide is a disease-modifying antirheumatic medication (DMARD). It is a non-biological isoxazole derivative with anti-inflammatory and immunomodulatory properties (Alldred and Emery 2001). Several studies have illustrated the anti-inflammatory and immunomodulatory effect of leflunomide in rheumatoid arthritis (Alldred and Emery 2001), multiple sclerosis (Rzagalinski et al. 2019), liver injury (Yao et al. 2004), dendritic cell function (Kirsch et al. 2005), and human T cell lines (Manna and Aggarwal 1999). In the body, it is converted to its active form, the metabolite A77-1726, also known as teriflunomide (Padda and Goyal 2021). The anti-inflammatory and immunoregulatory actions of leflunomide are related to its ability to suppress pro-inflammatory cytokines (Herrmann et al. 2000; Yao et al. 2003). Leflunomide inhibits the activation of NF-κβ, a critical pro-inflammatory transcription factor (Manna and Aggarwal 1999). Teriflunomide has also been shown to inhibit TNF-α-induced NF-κβ activation, a pro-inflammatory signaling pathway implicated in the pathophysiology of multiple sclerosis (Manna and Aggarwal 1999). In another study, Wei-Dong et al. showed that leflunomide inhibited the production of interleukin IL-1, IL-6, and TNF-α in peritoneal macrophages stimulated by lipopolysaccharide (Li et al. 2002). These properties of leflunomide suggest it is a promising chemical agent in ameliorating the AD.

The current study was designed to evaluate whether the anti-inflammatory and immunomodulatory effects of leflunomide could either ameliorate or even protect the neuropathological changes associated with AlCl_3_-induced AD in rats. Moreover, running hypothesis extended to explore the possible beneficial modulatory effects of leflunomide on one of the standard acetylcholinesterase inhibitors, rivastigmine, that widely used for AD treatment. Notably, AlCl_3_-induced AD rats’ model has predominantly been used and evoked pathological changes involve many symptoms of AD in human including cognitive decline, increase in β-amyloid and phospho-tau level, and amyloid plaque-like deposits. Al causes DNA injury in the brain by changing antioxidant enzymes or by binding to positively charged groups such as phosphates of DNA. Several studies showed that Al causes conformational changes of AβP and tau phosphorylation which results in the two hallmarks of AD in human: plaque deposits of the β-amyloid peptide Aβ and tau hyperphosphorylation (Pan et al. 2021; Kawahara and Kato-Negishi 2011).

## Materials and methods

### Chemicals, drugs, and kits

AlCl_3_ (molecular weight: 241.45 g/mol) was purchased from Alpha Chemika (Mumbai, India). Carboxy methyl cellulose (CMC) was provided by the El-Gomhouria Company for trading chemicals and medical appliances (Alexandria, Egypt). Leflunomide was obtained from Eva Pharma Industries (Alexandria, Egypt). Rivastigmine was procured from Novartis (Basel, Switzerland). Acetylcholinesterase activity assay kit (cat. no. MAK119) was supplied by Sigma Aldrich Chemical Company (St. Louis, MO, USA). β-Amyloid Aβ1–42 (cat. no. NBP2-69,916) and tau (cat. no. NBP2-81,164) ELISA kits were acquired from Novus Biologicals (Littleton, CO, USA). Three pro-inflammatory cytokines, NF-κβ (cat #: MBS453975), TNF-α (cat #: MBS824824), and IL-1β (cat #: MBS825017) ELISA kits, were bought from BioSource Inc. (San Diego, CA, USA).

### Experimental animals

Thirty-six male Wistar albino rats (180–250 g) were procured from Nile Company for Pharmaceutical and Chemical Industries (Cairo, Egypt). The rats were housed in a pathogen-free facility in cages with sawdust bedding (6 animals/cage) under standard conditions (12-h light/dark cycle, temperature range of 25 °C ± 2 °C, and relative humidity of 55% ± 5%, with water and food ad libitum) for a minimum of 1 week before the experiments for acclimatization and to ensure normal behavior and growth at the animal house in the Faculty of Medicine, Alexandria University (Alexandria, Egypt). The study protocol was approved by the Ethics Committee of the Faculty of Pharmacy, Damanhour University, (Damanhour, Egypt, approval no. 920PO21), and it is consistent with the Guide for the Care and Use of Laboratory Animals published by the National Institutes of Health (NIH Publications No. 8023, revised 1978). The experiments were conducted in the light time from 9:00 AM to 5:00 PM.

### Induction of the AD model

An AD-like model was induced in rats using aluminum chloride (AlCl_3_.6H_2_O) solution that was given daily using intraperitoneal route in three gradually ascending doses adopted from earlier literatures as follows: 50 mg/kg body weight/for 20 days (Chavali et al. 2020), 70 mg/kg body weight/for 20 days (Ali et al. 2016, 2022), and 100 mg/kg body weight/for 20 days (Justin Thenmozhi et al. 2015; Mohamed et al. 2021a, b; Zhao et al. 2020). These dose levels were selected to mimic AD stages (Ali et al. 2016) and to minimize the mortality rate.

### Experimental protocols

**Fig. 1:**
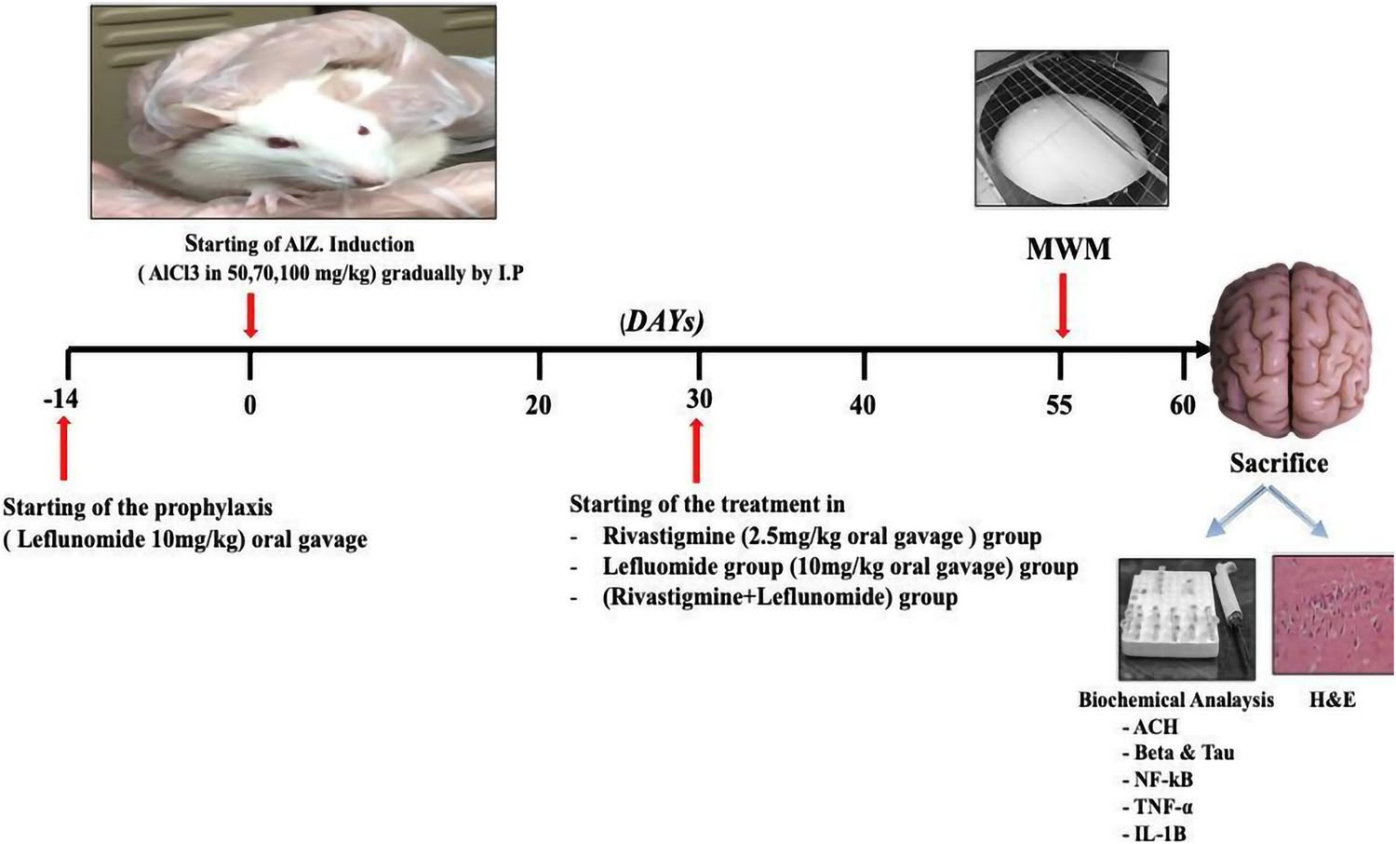
Experimental design

Figure 1 summarize experimental design in which thirty-six adult male albino rats were randomly assigned into two protocols for a total of six groups (*n* = 6 each) to receive one of the following regimens:

- **A) Treatment protocol**
  Thirty rats were included in the treatment protocol. All rats, except those in the control group, were assigned to AD induction using intraperitoneal daily dose of aluminum chloride for a period of 60 days in dose levels as aforementioned above. Treatment groups were as follows:
  - Group 1—control group that received vehicle 0.5% CMC aqueous solution was administered daily by oral gavage 3 days/week for 60 days.
  - Group 2—AD (AlCl3) that received intraperitoneal daily dose of aluminum chloride for a period of 60 days in dose levels as aforementioned above without any further treatment.
  - Group 3—reference group (Riva) that received rivastigmine 2 mg/kg (Akhtar et al. 2020) in 0.5% CMC aqueous solution administered daily by oral gavage starting from day 30 to day 60.
  - Group 4—experimental group (Lef) that received leflunomide 10 mg/kg (Jin et al. 2014; Kayhan et al. 2013) in 0.5% CMC aqueous solution administered by oral gavage 3 days/week starting from day 30 to day 60.
  - Group 5—combination group (Riva + Lef) that received combination of rivastigmine/CMC solution 2 mg/kg and leflunomide/CMC solution 10 mg/kg by oral gavage starting from day 30 to day 60.
    Starting different treatment regimens after 30 days of AlCl_3_ induction was intended to allow AlCl_3_ to accumulate in the brain and induce cognitive impairment.
- **B) Prophylaxis protocol**
  Prophylaxis group is a protection group that received leflunomide 10 mg/kg (Jin et al. 2014; Kayhan et al. 2013) in 0.5% CMC aqueous solution administered by oral gavage 3 days/week for 2 weeks before aluminum chloride induction and continued until the end of the experiment.

### Behavioral testing

#### Morris water maze (MWM) test

To assess spatial learning and memory in rats, the MWM test was tested in the last week of the study. The animals were directed to swim to a platform in a round pool (180 cm in diameter × 60 cm in height) containing water at 22 °C ± 2 °C filled to a height of 40 cm. The pool was divided by two imaginary perpendicular lines in the middle of the tank into four equal sections: north, south, east, and west. Throughout the project, a whiteboard 15 cm in diameter was submerged roughly 2 cm below the water’s surface in the center of one of the four sections. Starch powder was added to the water to make it opaque. Visual cues were adjusted outside the pool to help the rats locate the hidden platform.

The experimental protocol consisted of 4 training days and an additional probe trial day (Vorhees and Williams 2006). On each training day, a pseudorandom order of starting points was used but was the same for all animals. The starting location in each quadrant was maintained at the same position. The time to find the platform (escape latency) was recorded. Rats that failed to locate the platform within 120 s were placed on it for 15 s.

After the training session, the animals were returned to their home cages and allowed to rest for 24 h before the probe trial. The probe trial was a single 60-s trial in which the platform was removed entirely from the pool. The time spent in the target quadrant and the escape latency were all visually recorded.

### Biochemical testing and histopathological examination

#### Tissue sampling

The rats were fastened overnight, followed by thiopental overdose (50 mg/kg body wt.) (Helmy et al. 2014). The skull was opened carefully, and the whole brain of each rat was removed quickly and split into two halves mid-sagittally. According to the procedure listed earlier, the hippocampus was microdissected out from each half (Carleton et al. 1980); (1) one half was soaked with isotonic saline, dried out on filter paper, weighed, and quickly homogenized in ice-cold phosphate-buffered saline (pH 7.4). The homogenate was centrifuged at 2000–3000 rpm for 20 min at 4 °C. The supernatant was separated, kept at − 20 °C, and used for biochemical analyses. (2) The other hippocampal half was immediately fixed in 10% neutral buffered formalin for additional histopathological assessment by hematoxylin and eosin staining (Bazzari et al. 2019).

#### Biochemical measurement

##### Acetylcholinesterase activity assay kit

Rat hippocampal tissue homogenate was used to measure AChE activity with AChE assay kit according to the manufacturer’s guidelines. This assay is an optimized version of the Ellman method in which thiocholine, produced by AChE, reacts with 5,5′-dithiobis (2-nitrobenzoic acid) to form a colorimetric (412 nm) product, proportional to the AChE activity present.

##### Aβ1–42 and tau measurement

The hippocampal level of Aβ1–42 and tau protein was measured using rat ELISA kits according to the manufacturer’s protocol. Both the kits employ a sandwich ELISA procedure, and color change was measured spectrophotometrically at a wavelength of 450 nm. Their concentrations were calculated based on standards and are expressed in pg/mg of total protein.

##### Pro-inflammatory cytokine levels

Three pro-inflammatory cytokines, NF-κβ, TNF-α, and IL-1β, were assessed in the supernatant using ELISA kits according to the manufacturer’s instructions. The obtained values are presented in ng/mg and pg/mg.

#### Histopathological examination of the hippocampal tissues

Rat hippocampus brain samples were left for 24 h in 10% formalin and then soaked with water. Next, serial dilutions of alcohol were used to dry the samples. The samples were cleared in xylene and inserted in paraffin at 56 °C in a hot air oven for 24 h for light microscopy. Paraffin blocks were sectioned into 4-µm thickness, deparaffinized, and stained with hematoxylin and eosin (Ali et al. 2016). Standard light microscopy was used to examine the morphology of pyramidal neurons in the Cornu Ammonis zone 1 (CA1) region of the hippocampus. The number of normal pyramidal cells in the CA1 region of the hippocampus was used to determine the histological analysis of the neurons and the degree of hippocampal damage (Dhar et al. 2006). An observer blind to the group assignment counted the viable pyramidal neurons with blue-stained, intact round-shaped nuclei and without any nuclear fragmentation or karyopyknosis in the hippocampal CA1 subfield at a magnification of × 400.

### Statistical analysis

Statistical analysis was performed using one-way analysis of variance (ANOVA) followed by Tukey post hoc test. All statistical analyses were done using GraphPad Prism version 8.0 (GraphPad Prism Software Inc., San Diego, CA, USA). The experimental data are represented as mean ± SD. The results were considered significant when *p* < 0.05.

## Results

### Effect on learning and memory in MWM during training days

**Fig. 2:**
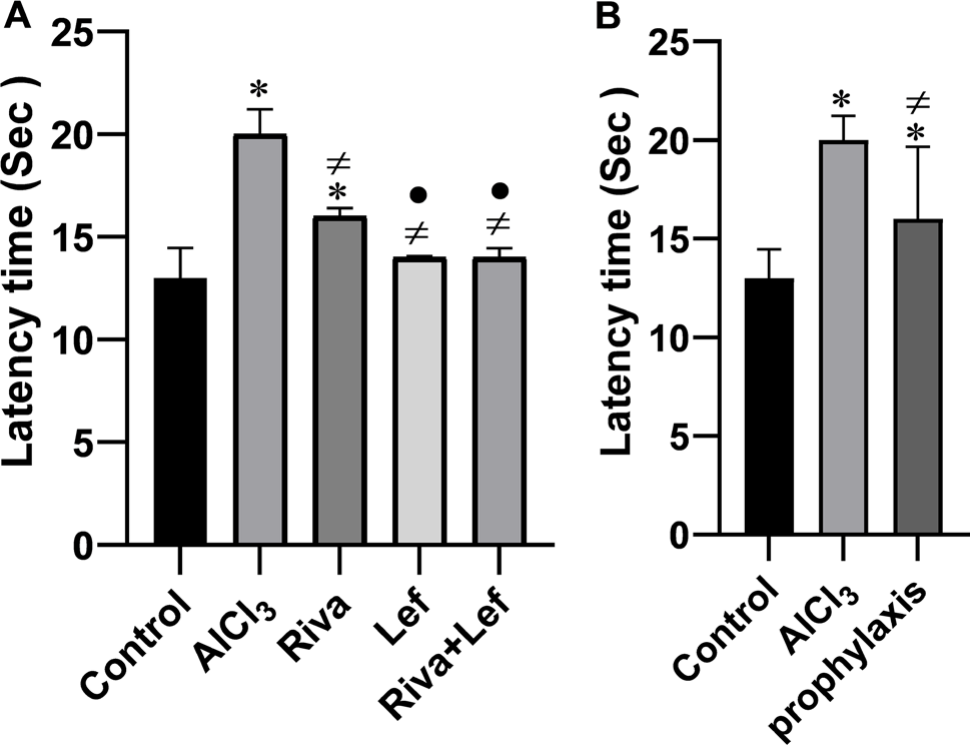
The effect of rivastigmine and/or leflunomide on mean latency time of Morris water maze test in AlCl_3_-induced AD in rats. **A** Treatment protocol, **B** prophylaxis protocol. Riva, rivastigmine; Lef, leflunomide. Data are presented as mean ± SD (*n* = 6) and tested by one-way ANOVA followed by the Tukey post hoc test using GraphPad Prism (v.8). Significant changes are reported at *p* < 0.05. *Significance relative to the control group. #Significance relative to AlCl_3_ group. •Significance relative to rivastigmine group

Figure 2A and B represent spatial learning of the different experimental groups in the MWM test. The results revealed that the average escape latency was significantly (*p* < 0.05) increased in the AlCl_3_ group compared to the control group. Leflunomide administration improved the ability of rats to reach the platform with an escape latency approaching the normal values during the training days compared to rivastigmine alone (Fig. 2A). These actions were similar to when leflunomide was used in the treatment protocol alone or when it is combined with rivastigmine. Furthermore, the escape latency was significantly (*p* < 0.05) shorter when leflunomide was used in the prophylaxis protocol compared to the AlCl_3_ group (Fig. 2B)

### Effect on learning and memory in the MWM probe test

**Fig. 3:**
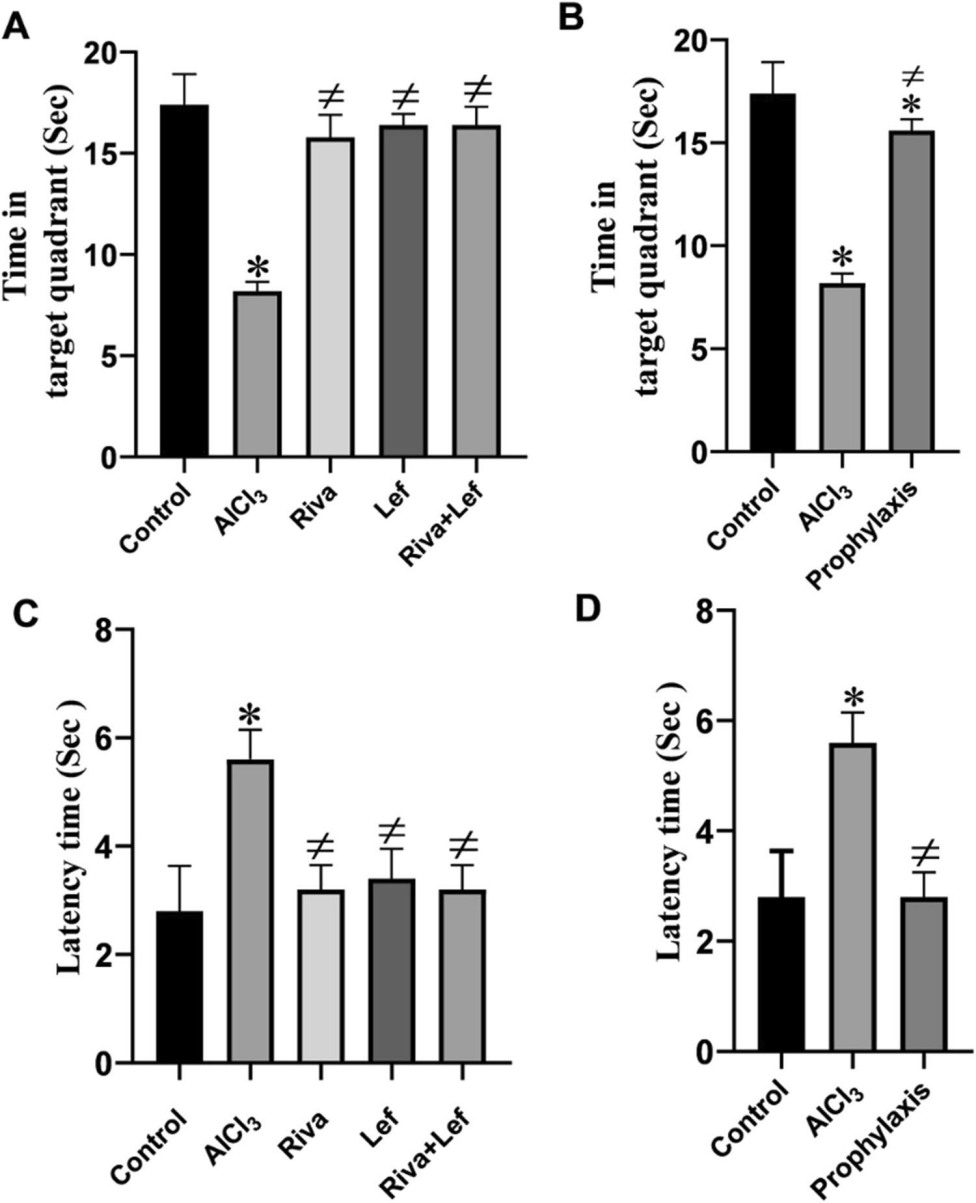
The effect of rivastigmine and/or leflunomide in the probe test of the Morris water maze test in AlCl_3_-induced AD in rats. **A** Time spent in the target quadrant for rats in the treatment protocol. **B** Time spent in target quadrant for rats in the prophylaxis protocol. **C** Latency time (treatment protocol). **D** Latency time of (prophylaxis protocol). Riva, rivastigmine; Lef, leflunomide. Data are presented as mean ± SD (*n* = 6) and tested with one-way ANOVA followed by the Tukey post hoc test using GraphPad Prism (v.8). Significant changes are reported at *p* < 0.05. *Significance relative to the control group. #Significance relative to the AlCl_3_ group

The probe test was performed without the platform and the latency to reach the target quadrant and the time spent in the target quadrant are presented in Fig. 3. The time spent in the target quadrant in the AlCl_3_ group was significantly (*p* < 0.05) less than those in the control group; however, treatment with rivastigmine and/or leflunomide in the treatment and prophylaxis protocol groups significantly (*p* < 0.05) increased the time spent in the target quadrant compared to that in the AlCl_3_ group (Fig. 3A and B).

In the same manner, the AlCl_3_ group significantly (*p* < 0.05) increased the escape latency time in the probe test compared to that of the normal group, events that were restored by rivastigmine and/or leflunomide in the treatment and leflunomide prophylaxis protocol (Fig. 3C and D).

### Effect on hippocampal AChE activity

**Fig. 4:**
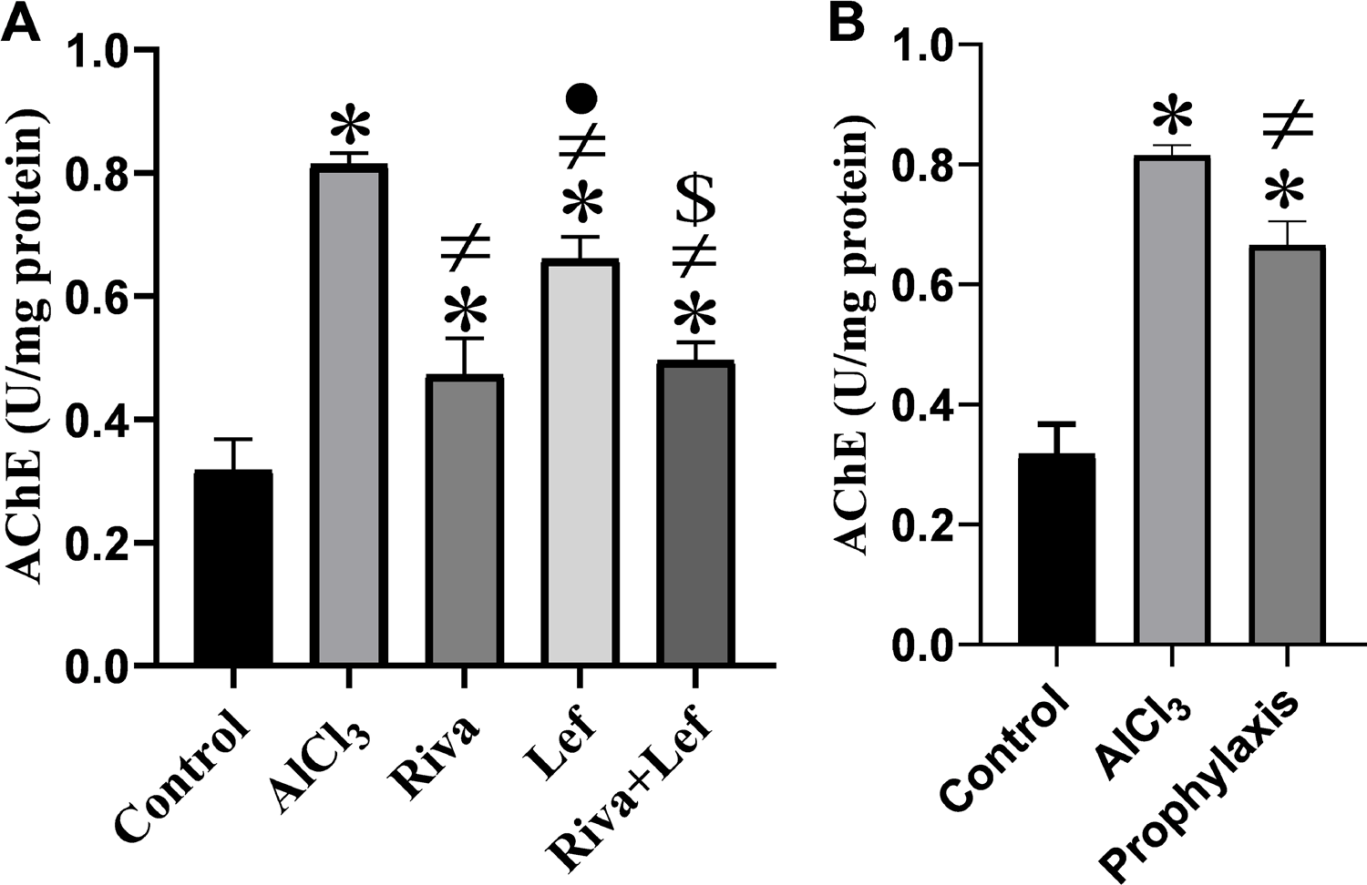
The effect of rivastigmine and/or leflunomide on hippocampal acetylcholinesterase (AChE) activity in AlCl_3_-induced AD in rats. **A** Treatment protocol, **B** prophylaxis protocol. Data are presented as mean ± SD (*n* = 6) and tested by one-way ANOVA followed by the Tukey post hoc test using GraphPad Prism (v.8). Riva, rivastigmine; Lef, leflunomide. Significant changes are reported at *p* < 0.05. *Significance relative to the control group. #Significance relative to the AlCl_3_ group. •Significance relative to the rivastigmine group. ^$^Significance relative to the leflunomide group

AlCl_3_ administration led to a significant (*p* < 0.05) rise in the rat hippocampal AChE activity as compared to the control group (Fig. 4). Nevertheless, rivastigmine and/or leflunomide in treatment as well as in leflunomide prophylaxis protocol significantly (*p* < 0.05) reduced the increased AChE activity as compared to the AlCl_3_ group (Fig. 4).

### Effect on hippocampal Aβ-amyloid and tau proteins levels

**Fig. 5:**
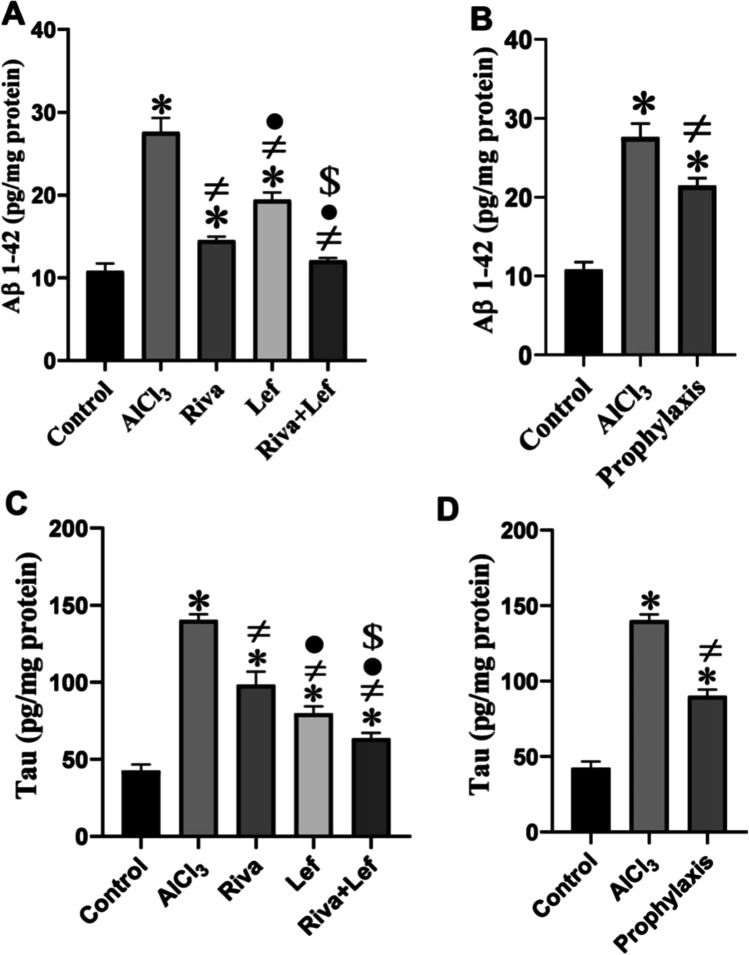
The effect of rivastigmine and/or leflunomide on hippocampal Aβ1–42 and tau proteins in AlCl_3_-induced AD in rats. **A** Aβ1–42 treatment protocol, **B** Aβ1–42 prophylaxis protocol, **C** tau treatment protocol, **D** tau prophylaxis protocol. Riva, rivastigmine; Lef, leflunomide. Data are presented as mean ± SD (*n* = 6) and tested by one-way ANOVA followed by the Tukey post hoc test using GraphPad Prism (v. 8). Significant changes are reported at *p* < 0.05. *Significance relative to the control group. #Significance relative to the AlCl_3_ group. •Significance relative to the rivastigmine group. ^$^Significance relative to the leflunomide group

AlCl_3_ significantly (*p* < 0.05) increased the brain concentrations of Aβ-amyloid and phosphorylated tau proteins compared to those in the control group (Fig. 5), while rivastigmine and/or leflunomide in the treatment or leflunomide prophylaxis protocol groups significantly (*p* < 0.05) decreased Aβ-amyloid and tau proteins in the brain level compared to those in the AlCl_3_ group.

### Effect on hippocampal pro-inflammatory cytokine levels

**Fig. 6:**
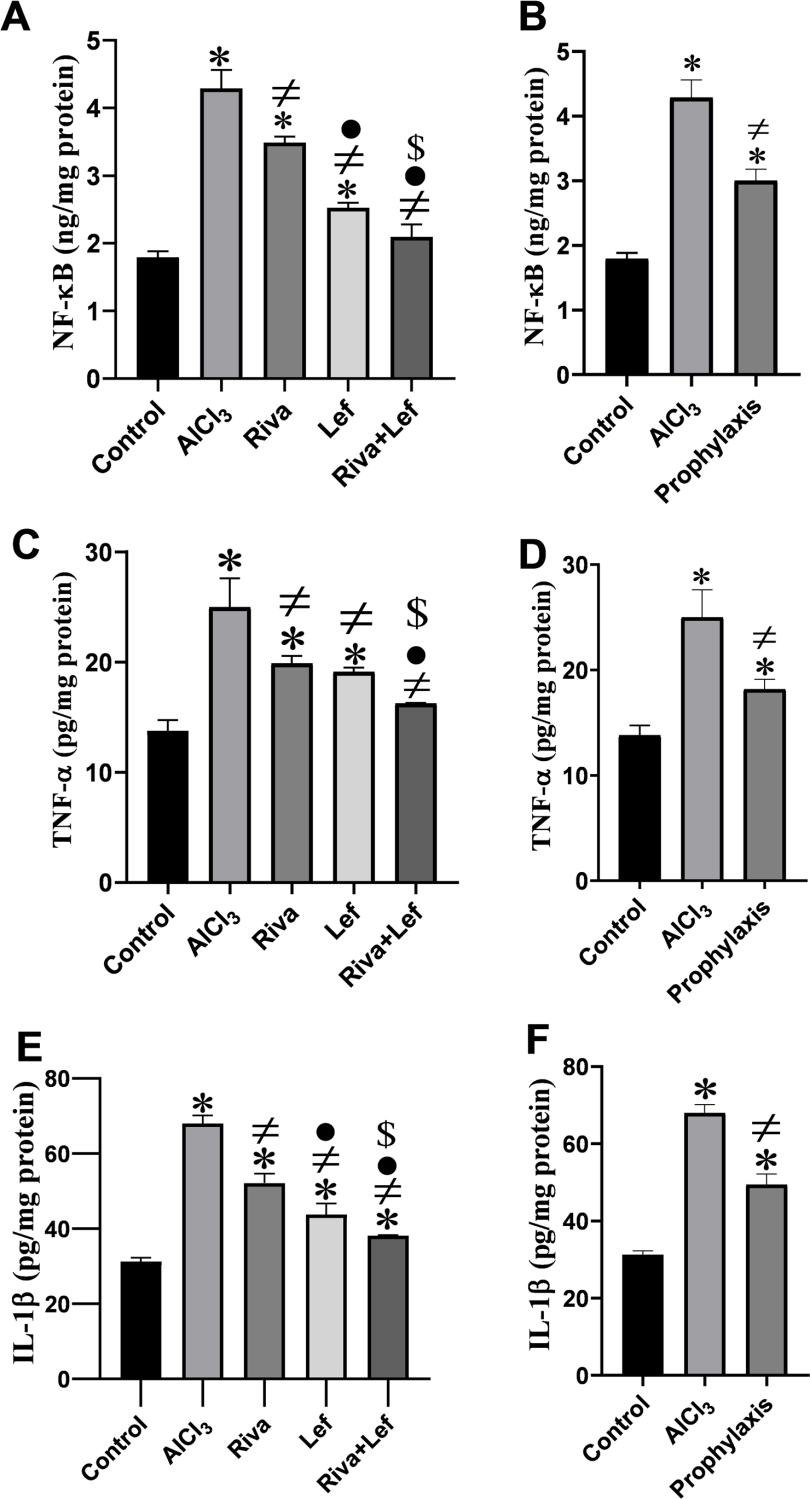
The effect of rivastigmine and/or leflunomide on hippocampal inflammatory cytokines in AlCl_3_-induced AD in rats. **A** NF-κB treatment protocol, **B** NF-κB prophylaxis protocol, **C** TNF-α treatment protocol, **D** TNF-α prophylaxis protocol, **E** IL-1B treatment protocol, **F** IL-1B prophylaxis protocol. Riva, rivastigmine; Lef, leflunomide. Data are presented as mean ± SD (*n* = 6) and tested by one-way ANOVA followed by the Tukey post hoc test using GraphPad Prism (v.8). Significant changes are reported at *p* < 0.05. *Significance relative to the control group. #Significance relative to the AlCl_3_ group. •Significance relative to the rivastigmine group. ^$^Significance relative to the leflunomide group

The level of NF-κβ, TNF-α, and IL-1β level in the hippocampus significantly (*p* < 0.05) increased in AlCl_3_ administration group compared to the control group (Fig. 6). On the other hand, rivastigmine and leflunomide in the treatment as well as the leflunomide prophylaxis protocol induced a significant (*p* < 0.05) decrease in pro-inflammatory cytokines in the hippocampus compared to the AlCl_3_ group. Moreover, the combined therapy of rivastigmine and leflunomide significantly (*p* < 0.05) diminished the levels of NF-κβ, TNF-α, and IL-1β compared to the AlCl_3_ group and their monotherapy groups (Fig. 6A and C).

### Histopathological examination of the rat hippocampus

**Fig. 7:**
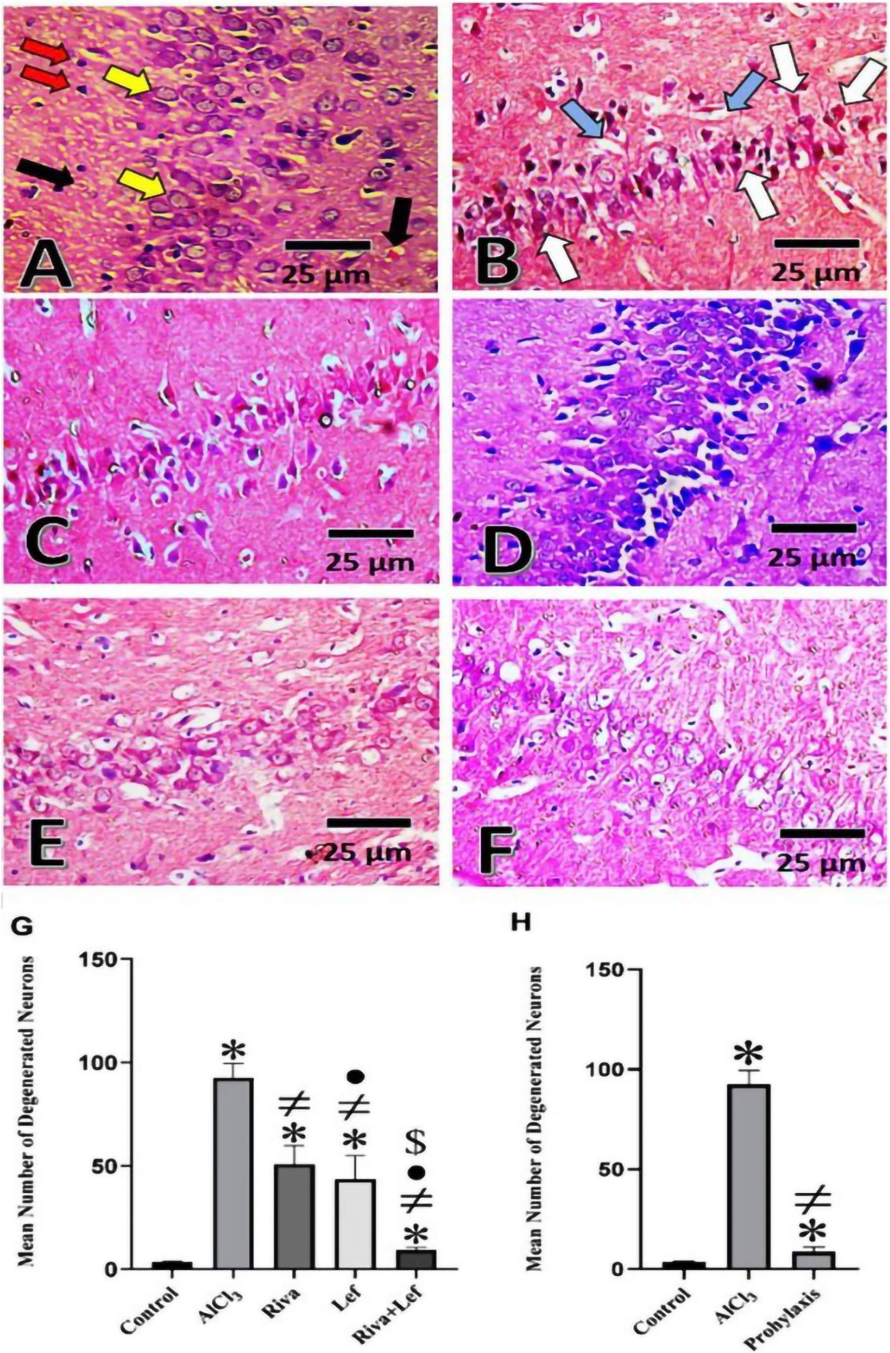
Histopathological changes and morphometric analysis of neurodegenerative changes in hematoxylin and eosin-stained hippocampus Sects. (40 × with scale bar 25 µm) from AlCl_3_-induced AD rats. Sections from **A** the control group; **B** AlCl_3_-induced AD (AlCl_3_); **C** the reference group (Riva) in the treatment protocol; **D** the experimental group (Lef) in the treatment protocol; **E** the combination group (Riva + Lef) in the treatment protocol; and **F** the prophylaxis protocol. **G** Morphometric analysis of the mean number of degenerated neurons ± SD (*n* = 6) in the therapeutic protocol and **H** the prophylaxis protocol (yellow arrow, normal pyramidal neuron; red arrow, normal glial cells; black arrow, neutrophil; white arrow, shrunken darkly stained pyramidal cells; and blue arrow, vacuolated cells)

Neuronal cells in the control group in the CA1 area (Fig. 7A) were arranged in 3–4 layers of closely packed small neurons with vesicular nuclei. There is a light eosinophilic neutrophil background with neuronal and glial cell processes and sparse neuroglial cells. However, the CA1 region of the hippocampus of AD (AlCl_3_ group) had the most pathogenic abnormalities, exhibiting a large number of deformed, darkly degenerating pyramidal neuron cells with lost nuclear features (Fig. 7B). Administration of rivastigmine and/or leflunomide in the treatment and leflunomide prophylaxis protocol improved the histopathological features with the restoration of the normal architecture pattern of CA1 hippocampal region (Fig. 7C–F). According to morphometric examination of degenerated neurons in the hippocampus, Alzheimer’s rats exhibited a considerable increase in the number of deteriorated neurons compared to the control group. On the other hand, rivastigmine and leflunomide in the treatment as well as leflunomide prophylaxis protocol induced a significant (*p* < 0.05) decrease in the number of degenerated neurons in the hippocampus than in the AlCl_3_ group. Moreover, the combined therapy with rivastigmine and leflunomide significantly (*p* < 0.05) diminished the number of degenerated neurons as compared to the AlCl_3_ group and each drug alone (Fig. 7G).

## Discussion

AD is a major brain cognitive disease that, to date, has no proven underlying mechanism(s) and its treatment remains one of the major neurological challenging. As a result, the high mortality and morbidity rates continue to increase (Zaher et al. 2019). There is growing evidence that inflammation may be a critical factor in AD development and exacerbation. Pro-inflammatory cytokines such as TNF-α, IL-1β, and IL-6 are elevated in the brains of people with AD, leading to the accumulation of Aβ plaque aggregates and tau hyperphosphorylation, resulting in neuronal loss (Kinney et al. 2018; Wang et al. 2015); thus, this study is conducted on decreasing NF-κβ which is a key mediator of these pro-inflammatory cytokines.

This study revealed that administration of rivastigmine and/or leflunomide in AlCl_3_-induced AD model in rats improved their spatial learning behavior, and significantly attenuated AChE activity and hippocampal pro-inflammatory cytokine release. Moreover, leflunomide prevented the development of amyloid plaque and tau protein expression provoked by AlCl_3_, suggesting a neuroprotective of leflunomide against AlCl_3_-induced AD model.

The behavioral changes demonstrated in the MWM test showed a significant increase in latency time, and the rats spent a shorter amount time in the target quadrant in AlCl_3_-induced AD rats compared to normal rats. These results reflect a decline in spatial learning and memory, demonstrating that AlCl_3_ is a neurotoxin, and its elevation in the brain is associated with cognitive impairment and dementia (Mohamed et al. 2021a, b). Furthermore, these findings are in consistent with earlier experimental studies in rats (Ahmad Rather et al. 2018; Justin-Thenmozhi et al. 2018).

*Interestingly*, the reduced cognitive functions in the AlCl_3_ group could be a result of reported major rise of AChE activity in the hippocampus compared to normal control rats. The activity of AChE in AD model rats has been reported to either decreased or increased. This could be attributed to differences in animal models, experimental methodologies, and sample collection times (Xiao et al. 2011). Similar to our study results, previous research has reported elevated AChE activity in AlCl_3_-exposed rats (Ahmad Rather et al. 2018; Lin et al. 2015; Prema et al. 2016; Qusti 2017). The increased AChE activity could be due to direct neurotoxic action AlCl_3_, which alters the kinetic properties of AChE (Zatta et al. 1994). Furthermore, it could possibly be due to IL-1β overexpression, which enhances AChE activity and production, as revealed in the current study (Schliebs et al. 2006). Long-term Al exposure causes APP gene overexpression and consequently Aβ production (Arendt et al. 1984), which elevates AChE activity through alpha7-nicotinic acetylcholine receptors (Fodero et al. 2004).

Although the elevation of AChE is a non-specific marker of disrupted cholinergic function as it is located both on cholinergic presynaptic and on non-cholinergic postsynaptic elements, however, it is worthy here to denote that the elevation of AChE together with both decline of spatial learning and memory and apparent histopathological loss of non-cholinergic postsynaptic neurons represented in the hippocampal tissues of AlCl_3_-induced AD rats’ model refers to disrupted cholinergic system. Indeed, the later histopathological changes revealed potential deficit in cholinergic inputs and loss of basal cholinergic neurons, which have axonal terminals in the hippocampus. However, most of the currently available medical treatments for AD focus on elevating acetylcholine levels by preventing its breakdown by acetylcholine esterase (AChE) (de Wilde et al. 2011). Indeed, many research shows that administering AChE inhibitors to AD patients can elevate acetylcholine levels and provide some symptoms relief (Lanctôt et al. 2003; Tariot 2006). The loss in cholinergic transmission could be correlated to the upstream disruption in the enzyme choline acetyltransferase (ChAT) which is responsible for synthesizing ACh, and the vesicular acetylcholine transporter (VAChT) uptakes the neurotransmitter into synaptic vesicles, which are involved in AD pathogenesis (Davies and Maloney 1976; Ozturk et al. 2006) and should be considered in future investigations. The inhibition of ChAT activity in the development of AD has further supported the idea that β-amyloid oligomers suppress the activity of ChAT (Nunes-Tavares et al. 2012; Winick-Ng et al. 2016). Spatial learning and memory have been linked in rat studies with ChAT activity in the hippocampus (Hawley et al. 2015). Additionally, it has been asserted that overexpressing ChAT in a rat model of Alzheimer’s disease can enhance cognitive functions by raising acetylcholine levels (Shin et al. 2016).

Additionally, in this study, sub-chronic administration of AlCl_3_ to rats significantly elevated pro-inflammatory cytokines such as TNF-α and IL-1β levels in the hippocampus compared to control rats. Similar findings have been demonstrated in other works (Ahmad Rather et al. 2018; Cao et al. 2016; Qusti 2017; Ravi et al. 2018). Al stimulates glial cells (Campbell et al. 2002) which in turn release of pro-inflammatory mediators such as TNF-α and IL-1β (Cao et al. 2016). Furthermore, Al^3+^ stimulates the transcription factor NF-κβ, which boosts the inflammatory cascades (Lukiw et al. 2005; Verstraeten et al. 2008). Al also inhibits the phagocytosis of Aβ peptides via NF-κβ-mediated down-regulation of the “triggering receptor produced in myeloid cells 2” (TREM2), which leads to Aβ42 peptide buildup in the brain (Akiyama et al. 2000). Secondary mechanisms of Aβ toxicity include tau phosphorylation and microtubule networks collapse, which are crucial underlying events for neuronal death and AD development (Jangra et al. 2015). Importantly, Aβ, inflammatory stimuli, induce microglial for the production of further pro-inflammatory cytokines like IL-1β which increases the activity of kinases involved in tau phosphorylation and exacerbates the disease (Barron et al. 2017). Also, TNF-α, which is another pro-inflammatory cytokine, is overexpressed and lead to elevate pre-tangle-associated pT231 epitope (Janelsins et al. 2008). NF-κβ activation leads to tau pathology by increasing the expression of SET gene isoform 1, which is elevated in the brains of Alzheimer’s patients (Feng et al. 2017). Hence, NF-κβ is most likely the key upstream mediator of the neuronal abnormalities observed in this study, including Aβ accumulation, pro-inflammatory cytokine overexpression, and apoptosis activation.

In contrast, co-administration of rivastigmine and/or leflunomide with AlCl_3_ to rats in both the treatment and prophylaxis protocol significantly improved spatial learning behavior via the shorter escape latency time and longer time spent in the target quadrant in the MWM test. These MWM findings of rivastigmine are inconsistent with those of a previous study (Abdel-Aal et al. 2011). The mechanism of improved cognitive performance by rivastigmine could be attributed to its ability to inhibit acetylcholinesterase which significantly decreases hippocampal AChE activity compared to AlCl_3_-treated rats (Eldufani and Blaise 2019). In the same context, several studies showed that rivastigmine has an anti-inflammatory effect by its effect on nAchRs besides being inhibitor of acetylcholinesterase activity (Abdel-Aal et al. 2021; Ibrahimet al. 2018).

To our knowledge, this study is the first to assess the neuroprotective and therapeutic effect of leflunomide in an AlCl_3_-induced AD model. Moreover, it was the first of its kind to be combined with rivastigmine in the treatment protocol. The cognitive improvement effect with leflunomide might be due to the demonstrated decreased in AChE activity and hence improving cholinergic neurotransmission. Also, the decreased AChE activity with leflunomide might be due to its ability to lower IL-1β concentrations, as demonstrated in this study. As mentioned in a previous study, AD onset begins with the reduction of ACh (Giacobini et al. 2002). Therefore, AChE inhibition by leflunomide could have a neuroprotective effect in AD development. This is the first study to show a leflunomide inhibitory effect on AChE activity.

Additionally, this study disclosed that co-administration of rivastigmine and leflunomide with AlCl_3_ resulted in a significant decrease in hippocampal TNF-α and IL-1β levels compared to AlCl_3_ rats. Furthermore, leflunomide significantly decreased hippocampal TNF-α and IL-1β levels compared to rivastigmine. One explanation for the lower hippocampal TNF-α and IL-1β levels by rivastigmine administration is its inhibition of the NF-κβ pathways (Kamal et al. 2009), which was further demonstrated in this study.

Molecular explanation in hippocampal TNF-α and IL-1β level reduction by leflunomide administration is due to its capacity to inhibit of NF-κβ, which is a central pro-inflammatory transcription factor, and this provides the molecular basis for its anti-inflammatory and immunosuppressive effects (Manna and Aggarwal 1999). Moreover, the suppression of NF-κβ activation reduced Aβ accumulation and tau phosphorylation.

*Histopathological analysis* of hippocampi from different groups supported all of the findings in the current study. In contrast to control rats, hematoxylin and eosin staining of hippocampal tissue revealed areas of brain cell death and degenerative alterations in the AlCl_3_ group. Previous research showed similar results in AlCl_3_-induced AD models (Mohamed et al. 2021a, b; Rifaai et al. 2020; Saad El-Din et al. 2020). The improved histopathology outcomes with rivastigmine in this study are also consistent with earlier studies in an AlCl_3_-induced AD model (Anwar et al. 2021). To our knowledge, this is the first study to demonstrate a therapeutic and neuroprotective effect of leflunomide on the histopathology in an AlCl_3_-induced AD model. Furthermore, it’s combination with rivastigmine in the therapeutic protocol showed a beneficial outcomes. The histopathological alternation in the hippocampus may be explained by the observed biochemical change previously discussed.

## Conclusions

In the current study, leflunomide showed a therapeutic and neuroprotective effect in AlCl_3_-induced AD in rats by its ability to improve learning behavior, diminish Aβ and tau burden, decrease the hippocampal AChE activity, and hamper NF-κβ, TNF-α, and IL-1β concentrations. The anti-inflammatory effect of leflunomide in the current research was significant compared to that of rivastigmine alone. Their combination may be a promising therapy for treating AD. Confirmation of these effects in clinical trials in the future is recommended.

## Supplementary information

Below is the link to the electronic supplementary material.

Supplementary file1 (PZFX 487 KB)

Supplementary file2 (PZFX 678 KB)

Supplementary file3 (PZFX 735 KB)

Supplementary file4 (PZFX 154 KB)

## Data Availability

The authors confirm the availability of all required data and materials.

## References

[CR1] Abdel-Aal RA, Assi AA, Kostandy BB (2011). Rivastigmine reverses aluminum-induced behavioral changes in rats. Eur J Pharmacol.

[CR2] Abdel-Aal R, Hussein O, Elsaady R, Abdelzaher L (2021). Celecoxib effect on rivastigmine anti-Alzheimer activity against aluminum chloride-induced neurobehavioral deficits as a rat model of Alzheimer’s disease; novel perspectives for an old drug. Journal of Medical and Life Science.

[CR3] Ahmad Rather M, Justin Thenmozhi A, Manivasagam T, Dhivya Bharathi M, Essa MM, Guillemin GJ (2018). Neuroprotective role of Asiatic acid in aluminium chloride induced rat model of Alzheimer’s disease. Front Biosci (schol Ed).

[CR4] Akhtar A, Bishnoi M, Sah SP (2020). Sodium orthovanadate improves learning and memory in intracerebroventricular-streptozotocin rat model of Alzheimer’s disease through modulation of brain insulin resistance induced tau pathology. Brain Res Bull.

[CR5] Akiyama H, Arai T, Kondo H, Tanno E, Haga C, Ikeda K (2000). Cell mediators of inflammation in the Alzheimer disease brain. Alzheimer Dis Assoc Disord.

[CR6] Ali AA, Ahmed HI, Abu-Elfotuh K (2016) Modeling stages mimic Alzheimer’s disease induced by different doses of aluminum in rats: focus on progression of the disease in response to time. of, 11, 2

[CR7] Ali AA, Khalil MG, Abd El-Latif DM, Okda T, Abdelaziz AI, Abu-Elfotuh K, Wahid A (2022) The influence of vinpocetine alone or in combination with Epigallocatechin-3-gallate, Coenzyme COQ10, Vitamin E and Selenium as a potential neuroprotective combination against aluminium-induced Alzheimer’s disease in Wistar Albino Rats. Arch Gerontol Geriatr 98:104557. 10.1016/j.archger.2021.104557 10.1016/j.archger.2021.104557 34706318

[CR8] Alldred A, Emery P (2001). Leflunomide: a novel DMARD for the treatment of rheumatoid arthritis. Expert Opin Pharmacother.

[CR9] Anuradha U, Kumar A, Singh RK (2022). The clinical correlation of proinflammatory and anti-inflammatory biomarkers with Alzheimer disease: a meta-analysis. Neurol Sci.

[CR10] Anwar HM, Georgy GS, Hamad SR, Badr WK, El Raey MA, Abdelfattah MAO, Sobeh M (2021) A Leaf Extract of Antioxidants (Basel). 10(6). 10.3390/antiox10060947 10.3390/antiox10060947 PMC8230640 34208063

[CR11] Arendt T, Bigl V, Tennstedt A, Arendt A (1984). Correlation between cortical plaque count and neuronal loss in the nucleus basalis in Alzheimer’s disease. Neurosci Lett.

[CR12] Aupperle KR, Bennett BL, Boyle DL, Tak P-P, Manning AM, Firestein GS (1999). NF-κB regulation by IκB kinase in primary fibroblast-like synoviocytes. J Immunol.

[CR13] Barron M, Gartlon J, Dawson LA, Atkinson PJ, Pardon M-C (2017). A state of delirium: deciphering the effect of inflammation on tau pathology in Alzheimer's disease. Exp Gerontol.

[CR14] Bazzari FH, Abdallah DM, El-Abhar HS (2019). Chenodeoxycholic acid ameliorates AlCl3-induced Alzheimer’s disease neurotoxicity and cognitive deterioration via enhanced insulin signaling in rats. Molecules.

[CR15] Campbell A, Yang EY, Tsai-Turton M, Bondy SC (2002). Pro-inflammatory effects of aluminum in human glioblastoma cells. Brain Res.

[CR16] Cao Z, Yang X, Zhang H, Wang H, Huang W, Xu F, Li Y (2016) Aluminum chloride induces neuroinflammation, loss of neuronal dendritic spine and cognition impairment in developing rat. Chemosphere 151:289-295. 10.1016/j.chemosphere.2016.02.092 10.1016/j.chemosphere.2016.02.092 26946116

[CR17] Carleton HM, Drury RAB, Wallington EA (1980). Carleton's histological technique.

[CR18] Chavali VD, Agarwal M, Vyas VK, Saxena B (2020). Neuroprotective effects of ethyl pyruvate against aluminum chloride-induced Alzheimer’s disease in rats via inhibiting toll-like receptor 4. J Mol Neurosci.

[CR19] Cummings J, Lee G, Nahed P, Kambar MEZN, Zhong K, Fonseca J, Taghva K (2022). Alzheimer's disease drug development pipeline: 2022. Alzheimers Dement (n y).

[CR20] Davies P, Maloney AJF (1976). Selective loss of central cholinergic neurons in Alzheimer’s disease. The Lancet.

[CR21] de Wilde MC, Penke B, van der Beek EM, Kuipers AAM, Kamphuis PJ, Broersen LM (2011). Neuroprotective effects of a specific multi-nutrient intervention against Aβ 42-induced toxicity in rats. J Alzheimers Dis.

[CR22] Dhar A, Kaundal RK, Sharma SS (2006). Neuroprotective effects of FeTMPyP: a peroxynitrite decomposition catalyst in global cerebral ischemia model in gerbils. Pharmacol Res.

[CR23] Du X, Wang X, Geng M (2018). Alzheimer’s disease hypothesis and related therapies. Transl Neurodegener.

[CR24] Eldufani J, Blaise G (2019). The role of acetylcholinesterase inhibitors such as neostigmine and rivastigmine on chronic pain and cognitive function in aging: a review of recent clinical applications. Alzheimers Dement (n y).

[CR25] Feng Y, Li X, Zhou W, Lou D, Huang D, Li Y, Zhou W (2017) Regulation of SET gene expression by NFkB. Mol Neurobiol 54(6):4477-4485 10.1007/s12035-016-9967-2 27351675

[CR26] Fodero LR, Mok SS, Losic D, Martin LL, Aguilar MI, Barrow CJ, Small DH (2004) α7‐Nicotinic acetylcholine receptors mediate an Aβ1− 42‐induced increase in the level of acetylcholinesterase in primary cortical neurones. J Neurochem 88(5):1186-1193 10.1046/j.1471-4159.2003.02296.x 15009674

[CR27] Giacobini E, Spiegel R, Enz A, Veroff AE, Cutler NR (2002). Inhibition of acetyl- and butyryl-cholinesterase in the cerebrospinal fluid of patients with Alzheimer’s disease by rivastigmine: correlation with cognitive benefit. J Neural Transm (vienna).

[CR28] Hawley WR, Witty CF, Daniel JM, Dohanich GP (2015). Choline acetyltransferase in the hippocampus is associated with learning strategy preference in adult male rats. Behav Brain Res.

[CR29] Helmy MM, Helmy MW, Abd Allah DM, Zaid AMA, El-Din MMM (2014). Selective ETA receptor blockade protects against cisplatin-induced acute renal failure in male rats. Eur J Pharmacol.

[CR30] Heneka MT, Kummer MP, Latz E (2014). Innate immune activation in neurodegenerative disease. Nat Rev Immunol.

[CR31] Herrmann ML, Schleyerbach R, Kirschbaum BJ (2000). Leflunomide: an immunomodulatory drug for the treatment of rheumatoid arthritis and other autoimmune diseases. Immunopharmacology.

[CR32] Holmes C, Cunningham C, Zotova E, Woolford J, Dean C, Kerr S, Perry VH (2009) Systemic inflammation and disease progression in Alzheimer disease. Neurology 73(10):768-774. 10.1212/WNL.0b013e3181b6bb95 10.1212/WNL.0b013e3181b6bb95 PMC2848584 19738171

[CR33] Ibrahim AN, Attallah MI, Elnaggar RA (2018) “Combination of cholecalciferol and rivastigmine improves cognitive dysfunction and reduces inflammation in STZ induced Alzheimer’s model experimentally in rats.” Egypt J Basic Clin Pharm 8. 10.11131/2018/101369

[CR34] Jangra A, Kasbe P, Pandey SN, Dwivedi S, Gurjar SS, Kwatra M, Lahkar M (2015) Hesperidin and silibinin ameliorate aluminum-induced neurotoxicity: modulation of antioxidants and inflammatory cytokines level in mice hippocampus. Biol Trace Elem Res 168(2):462-471. 10.1007/s12011-015-0375-7 10.1007/s12011-015-0375-7 26018497

[CR35] Janelsins MC, Mastrangelo MA, Park KM, Sudol KL, Narrow WC, Oddo S, Bowers WJ (2008) Chronic neuron-specific tumor necrosis factor-alpha expression enhances the local inflammatory environment ultimately leading to neuronal death in 3xTg-AD mice. Am J Clin Pathol 173(6):1768-1782 10.2353/ajpath.2008.080528 PMC2626388 18974297

[CR36] Jin H, Piao SG, Jin JZ, Jin YS, Cui ZH, Jin HF, Li C (2014) Synergistic effects of leflunomide and benazepril in streptozotocin-induced diabetic nephropathy. Nephron Exp Nephrol 126(3):148-156. 10.1159/000362556 10.1159/000362556 24855017

[CR37] Justin Thenmozhi A, Raja TR, Janakiraman U, Manivasagam T (2015). Neuroprotective effect of hesperidin on aluminium chloride induced Alzheimer’s disease in Wistar rats. Neurochem Res.

[CR38] Justin-Thenmozhi A, Dhivya Bharathi M, Kiruthika R, Manivasagam T, Borah A, Essa MM (2018). Attenuation of aluminum chloride-induced neuroinflammation and caspase activation through the AKT/GSK-3β pathway by hesperidin in wistar rats. Neurotox Res.

[CR39] Kamal MA, Greig NH, Reale M (2009). Anti-inflammatory properties of acetylcholinesterase inhibitors administered in Alzheimer’s disease. Anti-Inflamm Anti-Allergy Agents Med Chem (Formerly Curr Med Chem-Anti-Inflamm Anti-Allergy Agents).

[CR40] Kawahara M, Kato-Negishi M (2011). Link between aluminum and the pathogenesis of Alzheimer’s disease: the integration of the aluminum and amyloid cascade hypotheses. Int J Alzheimer’s Dis.

[CR41] Kayhan S, Guzel A, Duran L, Tutuncu S, Gunaydın M, Salis O, Selcuk MY (2013) Effects of leflunomide on inflamation and fibrosis in bleomycine induced pulmonary fibrosis in wistar albino rats. J Thorac Dis 5(5):641-649. 10.3978/j.issn.2072-1439.2013.09.20 10.3978/j.issn.2072-1439.2013.09.20 PMC3815717 24255778

[CR42] Kinney JW, Bemiller SM, Murtishaw AS, Leisgang AM, Salazar AM, Lamb BT (2018). Inflammation as a central mechanism in Alzheimer’s disease. Alzheimer's & Dementia: Transl Res Clin Interventions.

[CR43] Kirsch BM, Zeyda M, Stuhlmeier K, Grisar J, Smolen JS, Watschinger B, Säemann MD (2005) The active metabolite of leflunomide, A77 1726, interferes with dendritic cell function. Arthritis Res Ther 7(3):R694-703. 10.1186/ar1727 10.1186/ar1727 PMC1174963 15899055

[CR44] Lanctôt KL, Herrmann N, Yau KK, Khan LR, Liu BA, LouLou MM, Einarson TR (2003). Efficacy and safety of cholinesterase inhibitors in Alzheimer’s disease: a meta-analysis. CMAJ.

[CR45] Li W-D, Ran G-X, Teng H-L, Lin Z-B (2002). Dynamic effects of leflunomide on IL-1, IL-6, and TNF-alpha activity produced from peritoneal macrophages in adjuvant arthritis rats. Acta Pharmacol Sin.

[CR46] Lin WT, Chen RC, Lu WW, Liu SH, Yang FY (2015). Protective effects of low-intensity pulsed ultrasound on aluminum-induced cerebral damage in Alzheimer’s disease rat model. Sci Rep.

[CR47] Lukiw WJ, Percy ME, Kruck TP (2005). Nanomolar aluminum induces pro-inflammatory and pro-apoptotic gene expression in human brain cells in primary culture. J Inorg Biochem.

[CR48] Manna SK, Aggarwal BB (1999). Immunosuppressive leflunomide metabolite (A77 1726) blocks TNF-dependent nuclear factor-κB activation and gene expression. J Immunol.

[CR49] Mohamed EA, Ahmed HI, Zaky HS, Badr AM (2021). Sesame oil mitigates memory impairment, oxidative stress, and neurodegeneration in a rat model of Alzheimer's disease A pivotal role of NF-κB/p38MAPK/BDNF/PPAR-γ pathways. J Ethnopharmacol.

[CR50] Mohamed HE, Asker ME, Shaheen MA, Eissa RG, Younis NN (2021). Alleviation of fructose-induced Alzheimer’s disease in rats by pioglitazone and decaffeinated green coffee bean extract. J Food Biochem.

[CR51] Nunes-Tavares N, Santos LE, Stutz B, Brito-Moreira J, Klein WL, Ferreira ST, De Mello FG (2012). Inhibition of choline acetyltransferase as a mechanism for cholinergic dysfunction induced by amyloid-β peptide oligomers. J Biol Chem.

[CR52] Ozturk A, DeKosky ST, Kamboh MI (2006). Genetic variation in the choline acetyltransferase (CHAT) gene may be associated with the risk of Alzheimer’s disease. Neurobiol Aging.

[CR53] Padda IS, Goyal A (2021) Leflunomide. *StatPearls [Internet]* 32491731

[CR54] Perry VH, Cunningham C, Holmes C (2007). Systemic infections and inflammation affect chronic neurodegeneration. Nat Rev Immunol.

[CR55] Pan B, Xiaoting Lu, Han X, Huan J, Gao D, Cui S, Xiaofen Ju (2021). Mechanism by which aluminum regulates the abnormal phosphorylation of the tau protein in different cell lines. ACS Omega.

[CR56] Prema A, Thenmozhi AJ, Manivasagam T, Essa MM, Akbar MD, Akbar M (2016). Fenugreek seed powder nullified aluminium chloride induced memory loss, biochemical changes, Aβ burden and apoptosis via regulating Akt/GSK3β signaling pathway. PLoS ONE.

[CR57] Prinz M, Priller J, Sisodia SS, Ransohoff RM (2011). Heterogeneity of CNS myeloid cells and their roles in neurodegeneration. Nat Neurosci.

[CR58] Qusti SY (2017) Selenium and melatonin attenuates inflammation and oxidative stress in the brain of aged rats with aluminum chloride-induced Alzheimer. Int J Pharm Res Allied Sci 6(2)

[CR59] Rather MA, Khan A, Alshahrani S, Rashid H, Qadri M, Rashid S, Rehman MU (2021) Inflammation and Alzheimer’s disease: mechanisms and therapeutic implications by natural products. Mediators Inflamm 2021, 9982954. 10.1155/2021/9982954 10.1155/2021/9982954 PMC8352708 34381308

[CR60] Ravi SK, Ramesh BN, Mundugaru R, Vincent B (2018). Multiple pharmacological activities of Caesalpinia crista against aluminium-induced neurodegeneration in rats: relevance for Alzheimer’s disease. Environ Toxicol Pharmacol.

[CR61] Rifaai RA, Mokhemer SA, Saber EA, El-Aleem SAA, El-Tahawy NFG (2020). Neuroprotective effect of quercetin nanoparticles: a possible prophylactic and therapeutic role in alzheimer's disease. J Chem Neuroanat.

[CR62] Rzagalinski I, Hainz N, Meier C, Tschernig T, Volmer DA (2019). Spatial and molecular changes of mouse brain metabolism in response to immunomodulatory treatment with teriflunomide as visualized by MALDI-MSI. Anal Bioanal Chem.

[CR63] Saad El-Din S, Rashed L, Medhat E, Emad Aboulhoda B, Desoky Badawy A, Mohammed ShamsEldeen A, Abdelgwad M (2020). Active form of vitamin D analogue mitigates neurodegenerative changes in Alzheimer’s disease in rats by targeting Keap1/Nrf2 and MAPK-38p/ERK signaling pathways. Steroids.

[CR64] Schliebs R, Heidel K, Apelt J, Gniezdzinska M, Kirazov L, Szutowicz A (2006). Interaction of interleukin-1beta with muscarinic acetylcholine receptor-mediated signaling cascade in cholinergically differentiated SH-SY5Y cells. Brain Res.

[CR65] Shin K, Cha Y, Kim KS, Choi EK, Choi Y, Guo H, Kim YB (2016) Human neural stem cells overexpressing choline acetyltransferase restore unconditioned fear in rats with amygdala injury. Behavioural neurol, 2016 10.1155/2016/8521297 PMC4819097 27087745

[CR66] Shunan D, Yu M, Guan H, Zhou Y (2021). Neuroprotective effect of Betalain against AlCl. Biomed Pharmacother.

[CR67] Sinyor B, Mineo J, Ochner C (2020). Alzheimer's Disease, Inflammation, and the Role of Antioxidants. J Alzheimers Dis Rep.

[CR68] Tak PP, Firestein GS (2001). NF-κB: a key role in inflammatory diseases. J Clin Investig.

[CR69] Tariot PN (2006). Contemporary issues in the treatment of Alzheimer’s disease: tangible benefits of current therapies. J Clin Psychiatry.

[CR70] Verstraeten SV, Aimo L, Oteiza PI (2008). Aluminium and lead: molecular mechanisms of brain toxicity. Arch Toxicol.

[CR71] von Bernhardi R, Tichauer JE, Eugenín J (2010). Aging-dependent changes of microglial cells and their relevance for neurodegenerative disorders. J Neurochem.

[CR72] Vorhees CV, Williams MT (2006). Morris water maze: procedures for assessing spatial and related forms of learning and memory. Nat Protoc.

[CR73] Wang WY, Tan MS, Yu JT, Tan L (2015) Role of pro-inflammatory cytokines released from microglia in Alzheimer’s disease. Ann Transl Med 3(10) 10.3978/j.issn.2305-5839.2015.03.49 PMC4486922 26207229

[CR74] Winick-Ng W, Caetano FA, Winick-Ng J, Morey TM, Heit B, Rylett RJ (2016). 82-kDa choline acetyltransferase and SATB1 localize to β-amyloid induced matrix attachment regions. Sci Rep.

[CR75] Xiao F, Li XG, Zhang XY, Hou JD, Lin LF, Gao Q, Luo HM (2011). Combined administration of D-galactose and aluminium induces Alzheimer-like lesions in brain. Neurosci Bull.

[CR76] Yao HW, Li J, Chen JQ, Xu SY (2004). Leflunomide attenuates hepatocyte injury by inhibiting Kupffer cells. World J Gastroenterol.

[CR77] Yao HW, Li J, Jin Y, Zhang YF, Li CY, Xu SY (2003). Effect of leflunomide on immunological liver injury in mice. World J Gastroenterol.

[CR78] Zaher MF, Bendary MA, Abd El-Aziz GS, Ali AS (2019). Potential protective role of thymoquinone on experimentally-induced Alzheimer rats. J Pharm Res Int.

[CR79] Zatta P, Zambenedetti P, Bruna V, Filippi B (1994). Activation of acetylcholinesterase by aluminium (III): the relevance of the metal species. NeuroReport.

[CR80] Zhao Y, Dang M, Zhang W, Lei Y, Ramesh T, Veeraraghavan VP, Hou X (2020). Neuroprotective effects of Syringic acid against aluminium chloride induced oxidative stress mediated neuroinflammation in rat model of Alzheimer’s disease. J Functional Foods.

